# Assessing the Operational Feasibility of Integrating Point-of-Care G6PD Testing into *Plasmodium vivax* Malaria Management in Vietnam

**DOI:** 10.3390/pathogens12050689

**Published:** 2023-05-08

**Authors:** Emily Gerth-Guyette, Huyen Thanh Nguyen, Spike Nowak, Nga Thu Hoang, Đặng Thị Tuyết Mai, Vũ Thị Sang, Nguyễn Đức Long, Mercy Mvundura, Nhu Nguyen, Gonzalo J. Domingo, Bùi Quang Phúc

**Affiliations:** 1 Diagnostics Program, PATH, Seattle, WA 98121, USA; 2 Vietnam Program, PATH, Hanoi 10080, Vietnam; 3 National Institute of Malariology, Parasitology and Entomology (NIMPE), Hanoi 10000, Vietnam

**Keywords:** vivax malaria, tafenoquine, primaquine, diagnostics, G6PD deficiency, hemolytic anemia

## Abstract

*Plasmodium vivax* cases represent more than 50% of a diminishing malaria case load in Vietnam. Safe and effective radical cure strategies could support malaria elimination by 2030. This study investigated the operational feasibility of introducing point-of-care quantitative glucose-6-phosphate dehydrogenase (G6PD) testing into malaria case management practices. A prospective interventional study was conducted at nine district hospitals and commune health stations in Binh Phuoc and Gia Lai provinces in Vietnam over the period of October 2020 to October 2021. The STANDARD™ G6PD Test (SD Biosensor, Seoul, Republic of Korea) was incorporated to inform *P. vivax* case management. Case management data and patient and health care provider (HCP) perspectives, as well as detailed cost data were collected. The G6PD test results were interpreted correctly by HCP and the treatment algorithm was adhered to for the majority of patients. One HCP consistently ran the test incorrectly, which was identified during the monitoring and resulted in provision of refresher training and updating of training materials and patient retesting. There was wide acceptability of the intervention among patients and HCP albeit with opportunities to improve the counseling materials. Increasing the number of facilities to which the test was deployed and decreases in the malaria cases resulted in higher per patient cost for incorporating G6PD testing into the system. Commodity costs can be reduced by using the 10-unit kits compared to the 25 unit kits, particularly when the case loads are low. These results demonstrate intervention feasibility while also highlighting specific challenges for a country approaching malaria elimination.

## 1. Introduction

*Plasmodium vivax* is the second-most predominant malaria parasite globally and accounts for 39% of cases in the World Health Organization’s (WHO) Southeast Asia region [1]. *P. vivax* can form hypnozoites which are dormant parasite stages in the liver that cause relapses of infection weeks to years after the primary infections. Curing a patient of *P. vivax* requires the administration of a combination therapy that treats both blood-stage and liver-stage parasites [2,3]. Liver-stage parasites are currently treated with 8-aminoquinoline drugs such as primaquine (PQ) and tafenoquine (TQ) which have the potential to cause hemolytic anemia, a serious side-effect, in glucose-6-phosphate dehydrogenase (G6PD)-deficient individuals [4,5]. Safe effective management of *P. vivax* cases requires addressing G6PD deficiency and ensuring patients with normal G6PD enzyme activity levels complete a multi-day regimen with PQ or receive a single day regimen of TQ [6,7].

In Vietnam, malaria continues to be a persistent public health problem despite declining caseloads. In 2019, nearly 4700 malaria cases were reported, of which 32.2% were *P. vivax* [8]. In 2021, the number of malaria cases nationwide decreased significantly with 467 cases reported, of which *P. vivax* cases represented over half of all cases [8]. In aligning with the WHO goals and strategies for the Regional Action Framework for Malaria Control and Elimination 2016–2020 in the Western Pacific, Vietnam aims to eliminate all malaria by 2030. The National Institute of Malariology, Parasitology, and Entomology (NIMPE), with support from the Regional Artemisinin-resistance Initiative and other partners, aims to strengthen strategies to meet Vietnam’s malaria elimination goals. These strategies can be bolstered with the inclusion and scale of routine testing for G6PD deficiency and the widespread provision of radical cure treatments, that can reduce relapse cases of *P. vivax* and can halt onward transmission of parasites. The Malaria National Strategic Plan for 2021–2025 emphasized the necessity of providing a safe radical cure for *P. vivax* patients [9].

There have been numerous studies of G6PD deficiency in Vietnam involving multiple ethnic groups, multiple provinces, and multiple G6PD test types. Modeling based on surveys done by the Malaria Atlas Project predict a G6PD deficiency prevalence of 8.9% [10,11]. Until recently, access to a safe and effective radical cure for *P. vivax* infection was limited in Vietnam. National guidelines for managing *P. vivax* cases states that all patients with *P. vivax* malaria older than 6 months should be given chloroquine for 3 days and a 14-day course of PQ unless they have G6PD deficiency or they are pregnant [12]. In the absence of testing for G6PD deficiency, some health care providers would not prescribe primaquine to avoid the risk of hemolysis, whereas some providers would prescribe primaquine with minimal follow-up for safety and adherence, resulting in patients either not having access to the benefits of a cure to *P. vivax*, or being exposed to the risks with minimal benefits.

Point-of-care (POC) diagnostics for G6PD deficiency are now available and have the potential to expand access to safe radical cures as well as enable significant progress towards malaria control and elimination goals. Integrating a point of care G6PD test in routine case management may help mitigate the risks of providing radical cure in Vietnam. Realizing the potential of point of care G6PD tests to do so will require a clear understanding of the challenges and transitions that routine malaria case management will need to undergo to adopt them successfully. Vietnam represents a country approaching elimination, which presents unique training, implementation, and cost implications in the incorporation of a new commodity into current malaria treatment and elimination strategies. This paper aims to demonstrate the first instance of using G6PD testing as part of malaria treatment and elimination strategies in Vietnam.

## 2. Methods

### 2.1. Study Objectives

The primary goal of this study was to assess the operational feasibility of integrating point-of-care G6PD testing into the management of *P. vivax* malaria as part of routine care. Operational feasibility was defined across multiple dimensions including:

Fidelity to the *P. vivax* diagnostic and treatment algorithm and degree to which patients are treated according to the revised algorithm that incorporates G6PD screening (See Table 1). Data on patient care, including malaria diagnosis, G6PD deficiency testing, and treatment were compared to the revised algorithm to understand the rate of adherence and where and why patient care deviated from the algorithm.

HCP proficiency in terms of their use and knowledge of the STANDARD G6PD test. HCPs were assessed at three different times using a standardized observation checklist and knowledge test.

Costs associated with introducing and integrating point-of-care G6PD testing into routine case management at commune health centers and district hospitals. Costs were compiled across multiple categories using routine health system data and study procurement receipts.

Perceptions of health care workers and patients on the use of point-of-care G6PD testing assessed through semi-structured interviews conducted at multiple time points.

Adoption of a quality assurance (QA) method. The G6PD test used in the study includes a set of high and low control reagents that can be used for QA and quality control purposes. Each facility was instructed to run the controls once per month, document the results, and report any abnormalities along with any other device malfunctions.

### 2.2. Ethics Statement

WCG Institutional Review Board (20202625) and the NIMPE ethics committee (Decision No. 818/QD-VSR Approval) approved this study. Written informed consent was obtained for all participants. Minors under 18 provided assent and written informed consent was obtained from parents/legal guardians.

### 2.3. Materials

**Figure 1:**
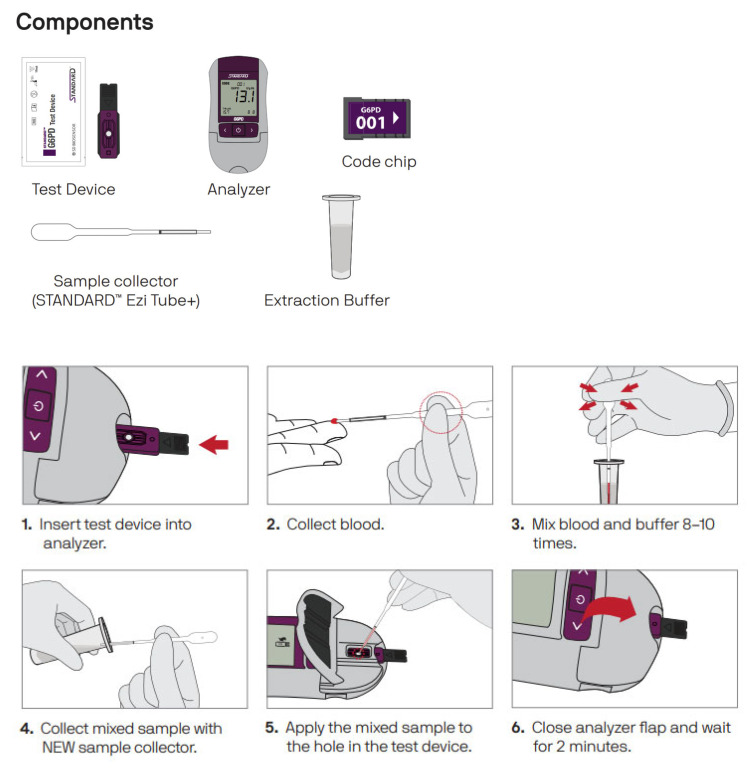
Test components and test procedure.

The STANDARD G6PD Test (SD Biosensor, Seoul, Republic of Korea) is an enzymatic colorimetric assay intended for the quantitative measurement of G6PD activity and total hemoglobin (T-Hb) concentration in finger-prick or venous whole blood. The STANDARD G6PD Test provides a numeric measurement of G6PD activity normalized by hemoglobin. This value can then be used in a semi-quantitative manner to classify individuals as G6PD normal, intermediate, or deficient according to established thresholds provided by the manufacturer. This classification can be used to inform clinical decision-making, particularly as it relates to priority applications, including informing treatments for *P. vivax* malaria. The STANDARD™ G6PD test has now been evaluated in multiple clinical studies [13,14,15,16,17]. Figure 1 shows the test components and workflow.

The disposable components of the test can be ordered in a kit containing test devices along with the required extraction buffer tubes and sample collectors. At the time of the study implementation, the test devices were only available in a 25-unit kit but since then, the manufacturer also offers a 10-unit kit.

### 2.4. Study Implementation

**Figure 2:**
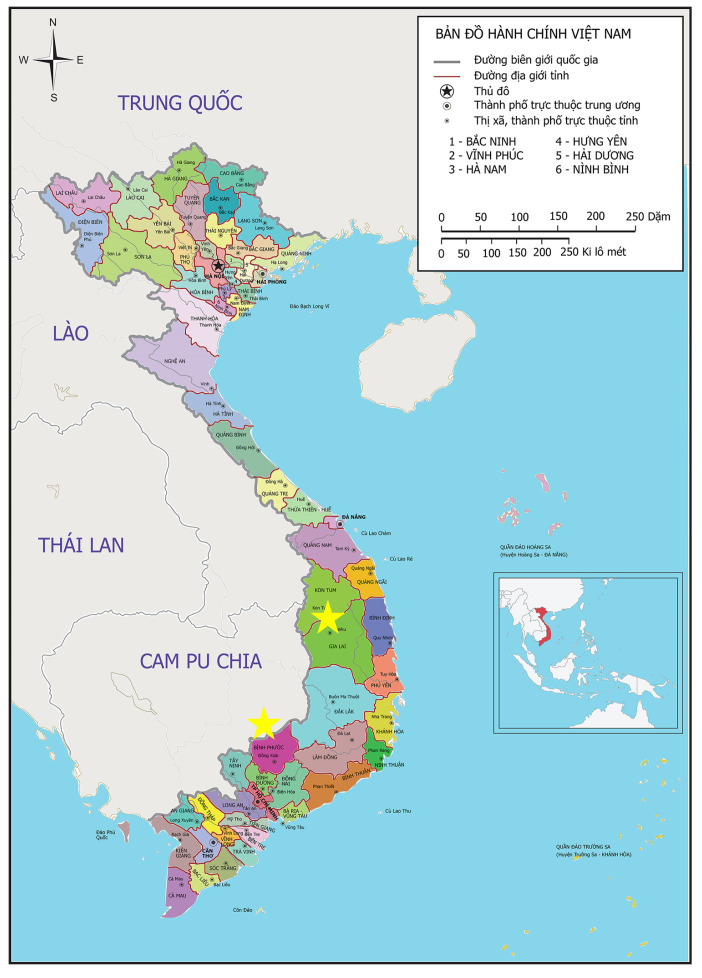
Map indicating the two provinces where the study was conducted: Binh Phuoc and Gia Lai provinces in Vietnam.

A prospective interventional study was conducted at nine district hospitals and commune health stations in Binh Phuoc and Gia Lai provinces in Vietnam (marked with a red star in Figure 2). Binh Phuoc and Gia Lai are among the five provinces with the highest number of malaria cases in Vietnam from 2012 to 2019 [18,19]. Binh Phuoc, a southern province, and Gia Lai, a province in the Central Highland have tropical monsoon climates with two seasons, rainy and dry [20,21].The communes and districts included in the study were selected based on recent *P. vivax* cases loads. The high-risk populations in these areas are forest goers, and farmers working on coffee, pepper, or cashew nut farms.

Prior to introduction of the G6PD test, a treatment algorithm was finalized (Table 1) through discussion with health officials and training on the use of the test and revised algorithm was conducted. The semi-quantitative G6PD value interpretation of the STANDARD G6PD test result was based on the current label at the time when the study was conducted. The training, provided by the study team and the national malaria program staff to the HCP involved in the studies, involved information on G6PD status, testing, and safe radical cure. HCP practiced the test with finger pricks from other trainees and control reagents. After a period of familiarization with the test, users were observed by a supervisor while running the test and certified as proficient in the use of the test. Finally, HCPs were trained in reporting test results, counseling patients on their G6PD status, using PQ, and monitoring for associated safety concerns.

Data were collected from October 2020 to October 2021. Data sources included augmented routine data on the test results and treatments provided to *P. vivax* patients. Fidelity to the algorithm was considered at each step of the case management process, including confirmation of *P. vivax* infection, use of the G6PD test, classification of the G6PD test result, and subsequent treatment prescription. HCP proficiency was assessed post-training (October 2020), midline (January 2021), and endline (October 2021). A standardized proficiency instrument was used to measure changes in proficiency and perceptions over time. Key informant interviews and focus group discussions were held with a subset of HCPs and *P. vivax* patients to explore the acceptability of G6PD testing, G6PD counseling, and PQ adherence messages, as well as barriers to seeking and completing treatment.

Cost data were collected throughout the intervention period to estimate the incremental financial costs to NIMPE of introducing and sustaining POC quantitative G6PD testing in routine care. These costs included the cost of the products and the costs associated with training and quality control activities. Economic costs to patients were not considered. The adoption of a QA method included the use of control reagents that support the STANDARD G6PD test. Each facility was instructed to run the controls once per month, document the results, and report any abnormalities along with any other device malfunctions. This QA method was assessed through monitoring visits, costing data, and interviews with the HCP who implemented it.

### 2.5. Data Analysis

Analysis population for the primary outcome included all patients who met the selection criteria and consented to their de-identified data being used in the study. Patient characteristics were presented using descriptive summary statistics. The distribution of G6PD test results was described according to the test instructions for use.

HCP assessments were conducted post-training (baseline) and at midline and endline sessions using standardized questionnaires what included questions about the G6PD test label, the testing procedure, and how to interpret the test results. HCPs were also asked to demonstrate the use of the test using control reagents. The questionnaires included a total of 26 questions. Participants who scored 85% or above (minimum of 22 questions correct) and were able to run the test with control reagents were considered proficient in the use of the test.

All quantitative analyses were carried out using Excel software. Continuous variables were described by their mean, standard deviation, median, quartiles 1 and 3, extreme values (minimum and maximum), and the number of missing data. Categorical variables were described by the total and percentage of each response and the number of missing data. Missing data were not included in the calculation of percentages. Given the sample size, the analysis was descriptive, and no statistical tests were performed.

Qualitative data from semi-structured interviews and focus group discussions were collected as part of in-person site visits and thus collected on-site (except for the endline data collection which was conducted online via Zoom software), recorded, and then later transcribed for analysis. The data were analyzed according to recurrent themes, participant type, and facility type.

### 2.6. Costing Analysis

An analysis of the financial costs of introducing G6PD testing at nine study facilities was conducted by examining the study G6PD diagnostic commodity and training costs, using the procurement costs incurred by the study. Exchange rates from the date of procurement of the commodities were used to calculate prices in US dollars. In addition, these costs were also analyzed using prices more likely to be incurred when commodities are procured via Global Fund procurement channels costs. We also evaluated the cost implications of procuring kits with smaller units: for the study, the units were procured as 25-unit kits. Since then, a 10-unit kit was made available by SD Biosensor and this analysis explores the implication of using 10-unit kits. Training costs for the training-of-trainers and the health facility-level trainings were estimated and annualized assuming training takes place every two years and the G6PD analyzers have a five-year lifespan. Training of health care workers was considered at the aggregate, annualized, and annualized per health facility level. The financial costs analysis at the nine study facilities was extended via a two-way sensitivity analysis to 25 scenarios representing differing malaria caseloads and differing number of health facilities with G6PD analyzers and trained users. These scenario costs were considered at a total health system financial cost level and a per patient level to illustrate financial costs for a health system to adopt G6PD testing. The estimated costs were multiplied by the number of malaria cases and health facilities accordingly. It was assumed that for every 100 facilities, at least one training-of-trainers will take place, that each facility would need to train two health care workers every two years, a 20% G6PD commodity buffer is needed, and the controls will be run five times per year at the facility. The distribution of *P. vivax* cases is assumed to be uniform across health facilities, as a simplifying assumption.

### 2.7. Impact of COVID-19

This study was conducted during acute periods of the COVID-19 pandemic which impacted the study in multiple ways. First, given the difference between previous rates of care-seeking, it is likely that lower than expected number of cases enrolled into the study due to lockdowns and reduced care-seeking. This was confirmed by anecdotal reports from the facility staff. Additionally, in order to comply with health guidance around regarding travel and large gatherings, some study activities were conducted remotely. All training sessions and the mid-term survey data collection were conducted in person at the selected districts. End-term assessment data collection was done online via Zoom software at each selected district hospital or commune health station.

## 3. Results

Between October 2020 and October 2021, 27 *P. vivax* confirmed patients were enrolled in the study along with 31 health care workers. This includes a subset of 6 patients and 24 HCP who participated in the qualitative data collection.

### 3.1. Fidelity to the P. vivax Diagnostic and Treatment Algorithm and Degree to Which Patients Are Treated According to the Revised Algorithm That Incorporates G6PD Screening

In total, there were 20 cases in Binh Phuoc Province and 7 cases in Gia Lai Province, see Table 2. This is inclusive of all confirmed *P. vivax* patients who were treated at the study facilities during that period, with the exception of a single patient at la MRơn commune health station, who was not enrolled in the study as the HCPs were focused on conducting COVID-19 prevention and control activities.

Most of the 27 patients were from ethnic minorities, including the S’tiêng (33.33%), the Gia Rai (22.22%), and a small number of people who were Khmer, M’nong, and Muong. Notably, most of the patients were recruited from two commune health stations in Binh Phuoc province with 10 patients treated at Đắk Ơ and 9 patients treated at Bu Gia Map facilities.

**Figure 3:**
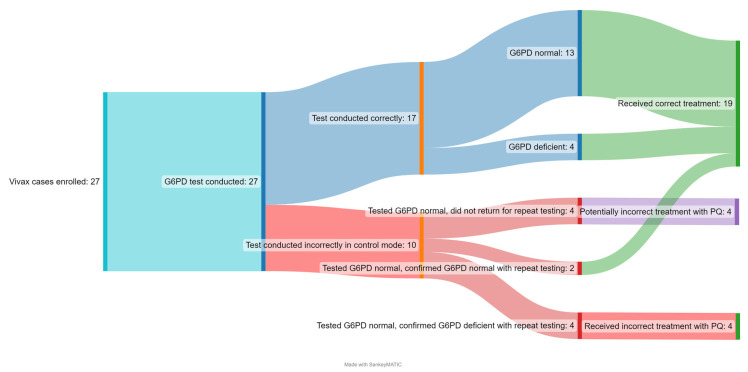
*P. vivax* patient cascade.

Figure 3 shows the patient cascade from G6PD testing to result classification to treatment prescription. All 27 confirmed *P. vivax* patients were screened for G6PD deficiency prior to treatment prescription. Ten patients in the Đắk Ơ commune health station were incorrectly tested by a single user, who had set the G6PD test set in control mode, which is used for testing control reagents, rather than testing mode, which is used for testing patient samples. Among the 17 patients that were tested correctly, 13 tested G6PD normal and 4 tested as G6PD deficient; all of them received the correct PQ treatment based on their G6PD classification. The G6PD deficient cases constituted two M’nong males, one Gia Rai male, and one S’tiêng female. No participants tested G6PD intermediate. Of the four patients who correctly tested G6PD deficient, none of them received the 8-weekly treatment. As per national treatment guidelines, 8-weekly treatment can be provided only in health facilities capable of blood transfusion and these participants declined referral to this facility and thus were not provided any radical cure.

The 10 patients in Đắk Ơ commune health station that were incorrectly tested in control mode, rather than testing mode originally tested G6PD normal and received the standard dose of PQ. This was identified when NIMPE conducted a monitoring visit and checked the results stored in the analyzer and discovered the error. The patients were asked to come back and test again. Four patients were unable to be contacted again and six patients retook the test, of which two of them were confirmed G6PD normal and four of them tested as G6PD deficient. The details of this retesting are outlined in Supplemental Table S1. Further investigation of this event highlighted that the instrument default is in normal mode for testing clinical specimens and the instrument had to be set explicitly in control mode. The commune health staff were not able to monitor drug adherence or possible side effects but did not receive or observe any reports of severe adverse events during the study period and none of the presumed G6PD deficient cases that had tested normal in the control mode reported that they had experienced any hemolytic events.

Thus, out of a total of 27 patients, 19 (70%) received the correct treatment based on their G6PD status, 4 or 15% of patients incorrectly received the standard dose of PQ, and it is unknown whether 4 (15%) received the correct or incorrect treatment, as their original test result may be invalid and they did not return for any follow-up. HCPs were retrained and training materials were updated after this event to clarify that clinical specimens should not be run in control mode.

### 3.2. Health Care Provider Proficiency: Use and Knowledge of the STANDARD G6PD Test

**Figure 4:**
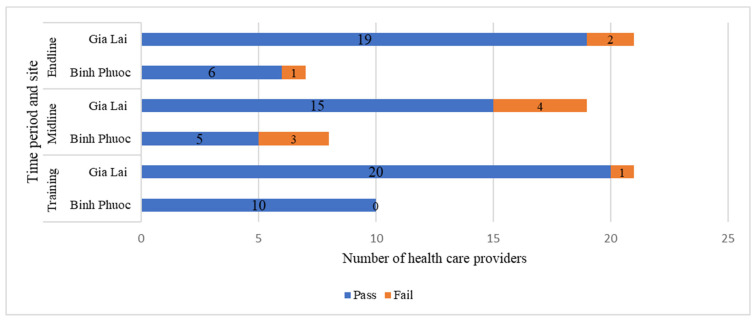
Health care provider proficiency scores over time.

HCP proficiency was assessed three times over the course of the study. In total, 31 HCPs from 9 health facilities in Binh Phuoc and Gia Lai provinces participated in the training including 3 managers from NIMPE, 13 doctors, 6 laboratory technicians, and 9 other care-giving health staff. At the midline assessment, 27 HCP were present, with 5 absences from the initial training and 1 additional doctor participating. At the endline assessment, 28 HCP participated, with 3 doctors absent from the original cohort. Nearly all participants (30/31) passed the proficiency test immediately after the training, with fewer participants (20/27) passing at the midline assessment and proficiency scores improving (25/28) again at the end of the study period (Figure 4). While fewer participants were included from Binh Phuoc as compared to Gia Lai, proficiency scores were comparable across the provinces.

### 3.3. Costs Associated with Introducing and Integrating Point-of-Care G6PD Testing into Routine Case Management at Commune Health Centres and District Hospitals

The key diagnostic test commodity costs in the study include the costs of the G6PD analyzer, the batteries for the analyzer, the test devices, and the G6PD control kit. Table 3 outlines the quantities and costs of testing supplies procured for the study using actual procurement costs recorded in the study and the likely costs if procured via Global Fund procurement channels. Total commodity purchase cost for G6PD diagnostics was USD 18,089 during the study, with the same costs estimated to be USD 15,707 when Global Fund procurement channels were used. Study procurement costs are higher at the per unit level due to the small-scale volume in the procurement.

The training of trainers (TOT) for the study cost USD 3120, or USD 1320 on an annualized basis, unless using Global Fund procurement prices for G6PD diagnostic commodities, in which case the annualized cost of the training-of the trainers is estimated to be USD 1143. The training of health care workers in the nine study facilities cost USD 3518 or USD 1759 on an annualized basis. This latter figure is estimated to be USD 1693 using Global Fund procurement prices for G6PD diagnostic commodities used as part of the training. Per facility training costs were estimated to be USD 195 with four health care workers trained per facility. A complete table of TOT costs can be found in the Supplemental Tables S2 and S3. For routine training outside of a study, it is assumed that only two health care workers per facility would be trained to use the G6PD analyzer and per facility this would cost USD 94 using Global Fund procurement costs.

### 3.4. Impact of Varying Deployment Strategies Impact on Intervention Costs

Table 4 displays the annual financial costs relevant to the health system for adopting G6PD testing in three categories: training, commodities, and QA. The costs in this are used to calculate different implementation scenarios. Estimated Global Fund procurement prices are used to better capture more realistic costs that the health system would encounter outside of a study. Training costs and device costs for the analyzer are amortized over two and five years, respectively.

To understand how deployment strategies in different malaria burden contexts impact, the financial costs of adopting G6PD tests sensitivity analyses were conducted on total and per patient financial costs based on varying caseloads (from 400 to 3200 cases) and health facilities (from 100 to 500) offering G6PD testing. In a scenario with a moderate malaria burden of 3200 cases and 500 health facilities offering G6PD testing, the estimated total financial cost to the health system to adopt G6PD testing is USD 164,741, or USD 51 per patient. In a scenario that reflects a context of malaria elimination, with 400 cases and 100 facilities, the estimated total financial cost to the health system to adopt G6PD testing is USD 32,929, or USD 82 per patient.

**Figure 5:**
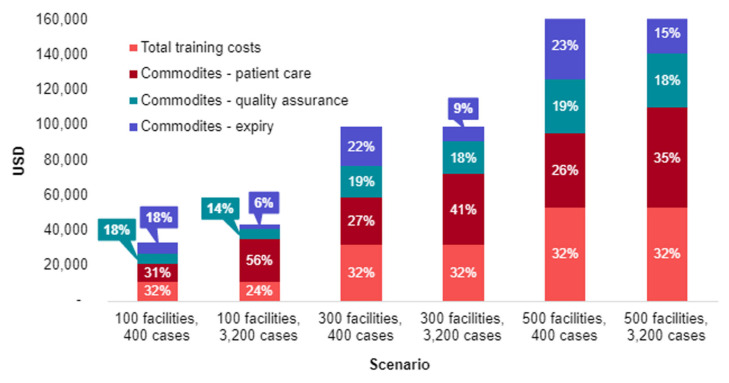
Total financial costs of different G6PD diagnostic implementation scenarios.

**Figure 6:**
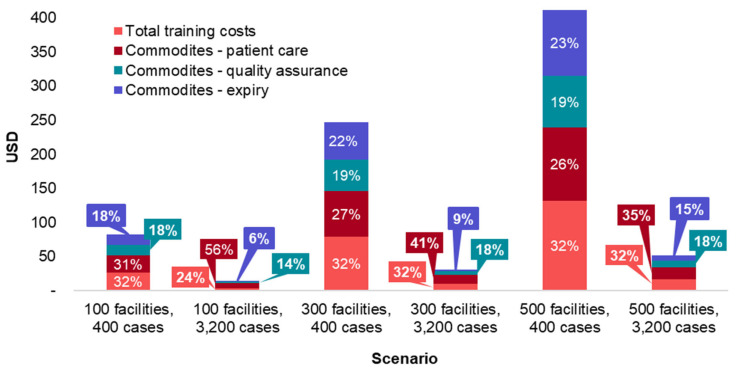
Total financial costs per patient of different G6PD diagnostic implementation scenarios.

To better understand which categories are driving the costs of different scenarios, the total costs of six scenarios were broken down by cost category (Figure 5). Commodities are displayed in three categories: (1) patient testing, which includes G6PD analyzers, G6PD devices, and auxiliary items, (2) QA, which includes G6PD devices and G6PD controls, and (3) expiry which only includes unused G6PD devices. It is assumed that the G6PD devices and controls have a one-year shelf life and devices not used will expire. Figure 6 shows the same data as per patient rather than total costs.

When considering total financial costs to the health system, as seen in Figure 5, the major cost driver is the number of health facilities with G6PD tests. Within each total cost scenario, regardless of the number of health facilities or malaria cases, training costs and patient care commodity costs are consistently the largest cost drivers. In the six scenarios, training costs as a percentage of total costs vary from 23% to 31% of total costs, while the same figures for patient care commodity costs are 28% to 57%.

**Figure 7:**
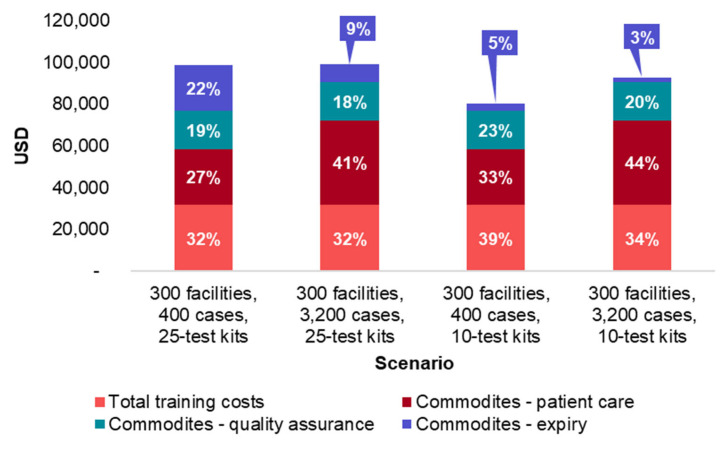
Total financial costs per patient of different G6PD diagnostic implementation scenarios with varying kit sizes.

Aside from the number of *P. vivax* cases and number of health facilities with a G6PD analyzer, the quantity of G6PD devices per box also has an impact on total financial costs to the health system due to costs associated with unused G6PD devices that expire. Figure 7 below displays four scenarios in which G6PD analyzers are deployed in 300 health facilities and cases vary from 400 to 3200, representing elimination and moderate burden settings, respectively. G6PD device kit quantities are also varied from the standard 25 devices per kit to the new 10 devices per kit. In moderate burden settings, switching from a 25-unit size box to a 10-unit box will reduce expiry costs by an estimated 71%, that is, from 9% of total financial costs to 3%, while the same switch in an elimination setting would reduce expiry costs by an estimated 83%, or from 22% to 5% of total financial costs.

### 3.5. Perceptions of Health Care Workers and Patients on the Use of Point-of-Care G6PD Testing

Qualitative data were collected through focus groups and interviews as part of the midline and endpoint data collection and key themes are presented below. A total of 7 interviews and 13 focus group discussions were conducted at two time points with a total of 24 HCP and 6 patients.

#### 3.5.1. Health Care Provider Background Knowledge of G6PD Deficiency

The majority of HCPs reported that they had some knowledge regarding G6PD deficiency and the associated risks. Most were able to explain that risk of G6PD deficiency stems from red blood cell destruction and leading to hemolysis. All HCPs agreed that the risks associated with G6PD deficiency should be prioritized in their facilities. All HCPs noted that they would have to transfer the patients to a higher-level facility if they had G6PD deficiency and needed a blood transfusion as none of the local health facilities offered that service. Only one participant, a doctor, described treating a G6PD deficient patient with side effects from primaquine including fatigue, darker color urine, and less amount of urine.

“There was a patient with *P. vivax* malaria back then. His condition was good but after the first dose of primaquine, then the second dose. The urine was less and darker in color. That was when I stopped giving that medicine to the patient…From our experience, when such symptoms appear…We stop using primaquine and give infusion for patients.” (Doctor, end-term assessment).

#### 3.5.2. Health Care Provider Perspectives on Training and the G6PD Test Implementation

All interviewees received training in October 2020. Overall, the interviewees agreed that the content and the length of the training were acceptable. Some participants recommended refresher training every 3 months, as they did not have many opportunities to perform the test due to the low volume of malaria patients. While most healthcare workers did not have difficulties using the test, one participant at Đắk Ơ commune health station ran ten patient specimens in control mode instead of sample mode, demonstrating some confusion between control and test procedures. Additionally, some of the participants thought the test was difficult to perform because of the following reasons: (1) having few patients and thus few opportunities to use the test, causing users to forget the steps required; (2) forgetting how to turn on and set up the devices; (3) having patients that refuse the test or hesitate to cooperate; and (4) not taking sufficient blood sample from the finger prick. Overall, HCPs’ willingness to use the G6PD test was high.

Most of the interviewees were satisfied with the QA testing, and the support and supervision they received from higher levels. The reasons provided included that the monthly control test reminder helped them remember the test procedure because sometimes there were too few patients for them to perform the test enough to remember the steps and that the supervision helped them answer questions and gain more hands-on experience. Some people suggested continuing to receive support such as training and providing them with more training materials.

HCPs in the study explained the purpose and benefits of the test to the patients and reported that while the time needed to run the test took 5–20 min, the time for consultation and explanation required more time, between 30 and 60 min in total. For patients with G6PD deficiency, some HCPs advised them to go to a higher level to get treatment, as the national treatment algorithm recommends treating deficient patients in locations where blood transfusion is available. Further, some HCPs reported that it was difficult for patients to understand the results of the test.

#### 3.5.3. Health Care Provider Testing Competency and Test Placement within the Health System

HCPs at hospitals and malaria program managers from district hospitals reported that the most appropriate staff to run the G6PD test are laboratory technicians because they are trained properly and have experience with similar assays. Others indicated that all cadres of HCPs (doctors, nurses, laboratory technicians, pharmacists, village healthcare staff) would be able to do it if trained. Although some people were against the idea of choosing commune health stations to conduct the test, most of the interviewees agreed to this, given that most malaria patients would go to the commune health stations for treatment first instead of the district hospitals. There was recognition that putting the test at lower-level facilities would serve to increase access to the test.

#### 3.5.4. Patient Perspectives on the Barriers and Facilitators to Taking Radical Cure Treatments

Some patients noted some difficulties in accessing diagnosis and treatment for malaria, including the distance from their location to the commune health station and language barriers. In general, patients took from 5 to 20 min to travel to and from the health facility to get treatment, and they spent about 30 to 60 min at the health facility. Regarding the advice they received from HCPs, one patient did not remember anything that the HCP said. Other patients recalled advice about drug treatment (number and types of tablets needed to take every day, the necessity of compliance), the importance of bed nets when sleeping, and healthy diets. One patient remembered being counseled not to take primaquine if they have G6PD deficiency. One patient did not understand the counseling from doctors, while the others said that it was understandable, and the doctors were willing to answer their questions. Most patients reported receiving the materials but did not read them. For G6PD deficient patients specifically, they were not aware of their condition prior to testing. All patients expressed reluctance to go to the district health centers for G6PD testing and radical cure treatment due primarily to the time required to travel.

### 3.6. Adoption of a Quality Assurance (QA) Method

Quality control (QC) tests results were recorded for 176 instances out of a total expected of 216 instances (9 health facilities × 12 months × 2 times of QC each month for low and high controls). Some facilities did the test more than once per month because errors occurred, or the results were out of range, and they re-ran the QC test while some health facilities were not able to run the control tests due to other required COVID-19 prevention and control tasks at the same time.

## 4. Discussion

This study aimed to demonstrate the feasibility integrating POC G6PD testing into *P. vivax* malaria management in Vietnam. Previously, the study sites in Binh Phuoc and Gia Lai Provinces recorded a fairly high number of *P. vivax* cases, at 159 cases in 2018 and 348 cases in 2019. However, during the study period, the number of cases decreased sharply, possibly reflecting the progression of the country towards elimination combined with the effects of the COVID-19 lock down measures during the study. Of the small number of patients, the majority of cases were male with only two females recruited. Albeit in the small sample size of this study (n = 27), the majority of those recruited represented ethnic minorities, who are more likely to be G6PD deficient, and disproportionately vulnerable to malaria infection.^11^ Indeed, the four G6PD deficient cases identified when the test was conducted correctly were of M’nong, Gia Rai, and S’tiêng origin.

The main results of the study demonstrate that testing is feasible and that patients, when tested correctly, receive the correct classification and subsequent treatment. The issue with a single user performing the test incorrectly in control mode demonstrates that particularly in an environment with low case volumes and few or fragmented users, adequate training and ongoing supervision are critical. In this case, the supervision systems established and carried about by NIMPE were largely successful in identifying and remedying problems. In this particular case, training materials were updated to emphasize that clinical specimens should not be run in control mode. However, the issue also demonstrates that even where training and supervision are conducted, user errors still occur, and follow-up of patients who receive radical cure treatment to monitor for adverse effects is still warranted. This can be achieved through both active follow-up and patient counseling, to educate patients on symptom monitoring.

The high scores for HCP proficiency post-training suggest that the training content is effective and that HCP at the commune level can learn how to use the moderately complex test. However, as the study progressed, proficiency scores declined and more HCPs failed the test, further reinforcing the need for ongoing training and supervision. User acceptability as well as proficiency for the STANDARD G6PD test has also been demonstrated in multiple settings across multiple end users [16,22,23,24].

The total costs of G6PD test integration are driven primarily by the number of health facilities that deploy G6PD tests. These data also show that per patient costs of adopting G6PD diagnostics increase as the number of malaria cases decreases; that is, per patient costs are expected to increase as a country moves towards an elimination scenario. Further, the use of the smaller kit, with 10 as opposed to 25 tests per box, can have a significant impact on reducing wastage and associated costs in percentage terms. While the savings in absolute monetary terms may not seem significant relative to overall malaria budgets, the perception that there is less waste and more efficiency may be important for promoting adoption among key decision-makers. This study did not investigate cost effectiveness of inclusion of G6PD testing. This has been investigated in settings with higher *P. vivax* caseloads where inclusion of G6PD testing was shown to be cost effective [22,23,24]. With the low case load in Vietnam, cost effectiveness is much harder to demonstrate and the overall costs for the intervention in context of overall malaria program costs are likely to be more critical.

Qualitative data show that knowledge of G6PD deficiency is strong among the target cadre of HCPs and their willingness to use the test was high. Infrequent use of the test among these workers was both cited and demonstrated to be a common challenge, pointing to the need for refresher training and ongoing supervision. The QA system of monthly QC testing could be one way to provide reinforcement regarding the test procedures but the proposed system of monthly testing in an elimination setting may result in more tests being used for QC than for patients. The frequency of QC testing and ways it supports QA will vary widely from setting to setting and country to country, based in part on national clinical laboratory guidelines. Interviewees reflected an ongoing debate about whether G6PD testing should be conducted by higher skilled clinicians and laboratory staff or front-line health care workers such as those at commune health stations, reflecting a potential tradeoff between ensuring quality testing or ensuring broad access. Again, with appropriate supervision, these results suggest that the test can be placed at the commune health station to ensure access to the test is available where patients seek care. Additionally, feedback from patients indicates that patient counselling processes can be improved, particularly among those who test G6PD deficient, to understand their options for treatment.

There are some limitations to this study; specifically, the low numbers of patients enrolled may limit the generalizability of findings. However, since the study, NIMPE scaled up the use of the STANDARD G6PD Test across the country. In 2022, the test was adopted in 33 health facilities. Since then, the Global Fund has approved NIMPE’s proposal to further scale the use of the G6PD test. The test will be expanded to 220 health facilities, including 3 institutes, 21 provincial centers, 71 district health centers, and 125 commune health stations. This rollout covers all high burden communes classified as having moderate risk of reintroduction or endemic malaria. NIMPE also developed additional training resources such as videos and job-aids and tools to support the broader intervention including materials for treatment adherence and pharmacovigilance. Establishment of quantitative G6PD testing to support *P. vivax* case management also prepares the program for adoption of TQ which also may support elimination strategies.

## 5. Conclusions

This study shows the feasibility of using G6PD tests, both HCP and patient experiences, and demonstrates the associated costs of and changes to health system training and supervision systems that may be required to successfully integrate point of care G6PD testing into malaria case management. The experience in Vietnam, along with the tools and resources developed during implementation, provide a valuable example to other malaria control programs that may be considering expanding access to radical cure through G6PD testing, particularly other countries

## Figures and Tables

**Table 1:** Semi-quantitative interpretation of the G6PD value measured on the STANDARD G6PD test to inform *P. vivax* radical cure treatment.

Male			Female		
G6PD Value	Classification	PQ Regimen	G6PD Value	Classification	PQ Regimen
≤3.9 U/g Hb	Deficient	8 weeks	≤3.9 U/g Hb	Deficient	8 weeks
>4.0 U/g Hb	Normal	14 days	4.0–6.0 U/g Hb	Intermediate	14 days
			>6.0 U/g Hb	Normal	14 days

**Table 2:** Description of *P. vivax* patients by province.

	Binh Phuoc	Gia Lai	Total
Total Number (n) of Patients	20	7	27
Age (years)			
Mean (SD)	25.56 (10.75)	29.71 (13.05)	26.63 (11.54)
Range	9–50	17–60	9–60
Age categories, n (%)			
<15 years	3 (15)	0 (0)	3 (11.11)
≥15 years	17 (85)	7 (100)	24 (88.89)
Gender, n (%)			
Male	18 (90)	7 (100)	25 (92.59)
Female	2 (10)	0 (0)	2 (7.41)
Ethnicity, n (%)			
Kinh	7 (35.00)	1 (14.29)	8 (29.63)
S’tiêng	9 (45.00)	0 (0)	9 (33.33)
Gia Rai	0 (0)	6 (85.71)	6 (22.22)
Khmer	1 (5.00)	0 (0)	1 (3.70)
M’nong	2 (10.00)	0 (0)	2 (7.41)
Muong	1 (5.00)	0 (0)	1 (3.70)

**Table 3:** Total quantity and cost of G6PD diagnostics procured for the study.

Item	Packaging for Procurement	Quantity	Study Procurement Costs **		Likely Cost if Procured via Global Fund ***	
			Price per Package (USD)	Total Cost (USD)	Price per Package (USD)	Total Cost (USD)
SD Biosensor STANDARD G6PD Analyzer	Box of 1	14	480.10	6721	406.00	5684
Batteries	Box of 4 (AAA)	14	0.52	7	0.52	7
G6PD test devices	Box of 25	71	120.04	8523	101.50	7207
G6PD test devices ****	Box of 10	-	-	-	40.60	-
G6PD control kit	Box of 10 high and 10 low controls	69	41.00	2829	40.60	2801
Delivery fee *				8		8
Total cost				18,089		15,707

* Shipping fee of G6PD kits and controls to three districts: Bu Gia Map, Krong Pa, la Pa was 60,000 VND (2.59 USD) for each district. ** Study procurement prices in the table above include value added tax (VAT), distributor profit markups, and costs to ship the products from South Korea to NIMPE in Hanoi. *** The Global Fund procurement costs are estimated to be 16% more than factory gate product prices of the diagnostic products. The 16% figure was based on markups seen by United Nations Office for Project Services (the Global Fund procurement agent) for the procurement of malaria RDTs and obtained via correspondence. **** The ten-unit kit size was not available until 2023 and thus not used in this study. However, costs of this product were used as part of the cost analyses to understand how kit size drives costs and how smaller kits can impact wastage.

**Table 4:** Annual per unit financial costs for adopting G6PD testing in three categories: training, commodities, and quality assurance.

Category	Item	Amount in USD
Training	Training of trainers at one institute	1143
	Training for one health facility	94.03
Commodities	One G6PD analyzer	81.41
	One box of GG6P devices	101.50
	Auxiliary items per patient	0.06
Quality assurance	One box of quality controls	40.60

## Data Availability

The data presented in this study are available on request from the corresponding author. The data are not publicly available due to due to the small and highly focal study population wherein confidentiality may be at risk.

## Supplementary Materials

The following supporting information can be downloaded at: https://www.mdpi.com/article/10.3390/pathogens12050689/s1, Table S1: All participants tested multiple times with the STANDARD G6PD Test, Table S2: Total cost of training on G6PD testing for the training-of-trainers, Table S3: Total cost of training for G6PD testing for health workers at health facilities.
