# Supplementary material for: Assessing the Operational Feasibility of Integrating Point-of-Care G6PD Testing into Plasmodium vivax Malaria Management in Vietnam
Source: Pathogens. 2023 May 8;12(5):689. doi: 10.3390/pathogens12050689 (PMC10222310; doi:10.3390/pathogens12050689)
Supplement: Supplementary file 1 [file pathogens-12-00689-s001.zip › pathogens-2306424-supplementary.pdf]

Supplemental Tables

1. All participants tested multiple times with STANDARD G6PD Test
2. Costing table:

Supplemental Table 1. All participants tested multiple times with the STANDARD G6PD Test

ID	Province	District hospital/ commune health station	Age	Sex	G6PD test results			Reason for multiple tests	G6PD level
					1st time	2nd time	3rd time		
536	Binh Phuoc	Đắk Ơ[b]	33	Male	6.9	7.3	1.9	1st and 2nd time: control mode	Deficient
677	Binh Phuoc	Đắk Ơ[b]	20	Male	7.5	0.1	2.3	1st time: control mode, 2nd time: to ensure accuracy	Deficient
693	Binh Phuoc	Đắk Ơ[b]	21	Male	8.3	8,5	-	1st time: control mode	Normal
107	Binh Phuoc	Đắk Ơ[b]	32	Male	9.2	0.1	-	1st time: control mode	Deficient
290	Binh Phuoc	Đắk Ơ[b]	34	Male	8.6	4.6	-	1st time: control mode	Normal
302	Binh Phuoc	Đắk Ơ[b]	45	Male	7.9	0.1	-	1st time: control mode	Deficient
BGM/ G6PD /001	Binh Phuoc	Bu Gia Map[b]	14	Male	1.4	0.9		to ensure the result accuracy	Deficient
BGM/ G6PD /002	Binh Phuoc	Bu Gia Map[b]	9	Male	1.6	1.1		to ensure the result accuracy	Deficient

Costing Tables:

Using Global Fund procurement prices for G6PD diagnostic commodities the annualized cost of the training-of the trainers is estimated to be $1,143. The G6PD analyzers are assumed to be kept at the malaria institute and not used for routine case management, therefore their costs are included in the total training costs. The cost of the G6PD analyzer was amortized over 5 years, an estimate of the lifespan of the analyzer. All other costs are amortized over 2 years, an estimate of the training-of-trainer frequency. Other costs included in the training-of-trainer analysis were gloves, swabs, batteries, lunch, and a tea break.

Supplemental Table 2. Total cost of training on G6PD testing for the training-of-trainers

Item	Unit	Amount (supplies/ people)	Using study G6PD commodity procurement prices		Using likely Global Fund G6PD commodity procurement prices	
			Price per unit (USD)	Total cost (USD)	Price per unit (USD)	Total cost (USD)
Number of staff	Person	17				
G6PD analyzers	analyzer	5	480.10	2,401.00	406.00	2,030
G6PD test devices	Devices	3	120.04	360.00	101.50	305.00
G6PD test controls	Box of controls	3	41.00	123.00	40.60	122.00
Batteries	Box of 4 (AAA)	5	0.52	3.00	0.52	3.00
Gloves	box	1	4.33	4.00	4.33	4.00
Swabs	bag	1	5.18	5.00	5.18	5.00
Alcohol	Jar	1	2.99	3.00	2.99	3.00
Lunch	Per person	17	8.66	147.00	8.66	147.00
Tea break	Per person	17	1.73	29.00	1.73	29.00
Training materials for trainees	Per person	17	2.17	37.00	2.17	37.00
Certificate printing fee		17	0.43	7.00	0.43	7.00
**Total cost per TOT training**				**3,120.00**		**2,692.00**
**Total cost annualized (assuming TOT training occurs every 2 years, and G6PD analyzer has 5-year lifespan)**				**1,320.00**		**1,143.00**

Supplemental Table 3. Total cost of training for G6PD testing for health workers at health facilities

Item	Unit	Amount	Using study G6PD commodity procurement prices		Using likely Global Fund G6PD commodity procurement prices	
			Price per unit/person (USD)	Total cost (USD)	Price per unit/person (USD)	Total cost (USD)
**Both provinces (trainees = 36)**						
Lunch	Person	42	8.62	362.00	8.62	362.00
Tea break	Person	42	1.72	72.00	1.72	72.00

Training materials	Set	42	2.16	91.00	2.16	91.00
Certificate printing fee	Sheet (of paper)	36	1.73	62.00	1.73	62.00
Job aids	Person	18	4.31	78.00	4.31	78.00
G6PD test devices	G6PD devices kit (25 pieces)	7	120.04	840.00	101.50	711.00
G6PD controls	Control kit (20 pieces)	8	41.00	328.00	40.60	325.00
Gloves	box	3	2.88	9.00	2.88	9.00
Swabs	bag	3	5.16	15.00	5.16	15.00
Alcohol	Jar	3	2.97	9.00	2.97	9.00
Trainer travel costs	person	2	661.08	1,322.00	661.08	1,322.00
Trainee travel costs	person	18	4.31	78.00	4.31	78.00
Trainer per diem	person	2	126.09	252.00	126.09	252.00
**Total cost**				**3,518.00**		**3,385.00**
**Total cost annualized (training occurs every 2 years)**				**1,759.00**		**1,693.00**
**Total per health facility (n = 9) with 4 trainees - study training**				**195.00**		**188.00**
**Total per health facility (n = 9) with 2 trainees - routine training**				**98.00**		**94.00**
